# Triglyceride glucose index as a predictor for non-alcoholic fatty liver disease: insights from a longitudinal analysis in non-obese individuals

**DOI:** 10.3389/fmed.2024.1429413

**Published:** 2024-07-08

**Authors:** Qi Ning, Keyang Zheng, Jiafu Yan, Chao Zhu

**Affiliations:** ^1^Clinical Medical College, Yangzhou University, Yangzhou, China; ^2^Center of Hypertension, Beijing Anzhen Hospital, Capital Medical University, Beijing, China; ^3^Affiliated Hospital of Yangzhou University, Yangzhou University, Yangzhou, China

**Keywords:** triglyceride glucose index, nonalcoholic fatty liver disease, NAFLD, fatty liver index, non-obese population

## Abstract

**Background:**

A substantial portion of non-obese population is afflicted with Non-alcoholic Fatty Liver Disease (NAFLD). The Triglyceride Glucose (TyG) index, a quantifier of insulin resistance magnitude, is determined by the product of fasting plasma glucose and triglyceride concentrations. The relationship between the TyG index and NAFLD within this cohort remains ambiguous.

**Methods:**

We conducted a retrospective analysis utilizing datasets acquired from the Dryad digital repository. Non-obese participants (BMI < 25 kg/m^2^) were enrolled at the Wenzhou Medical Center of Wenzhou People’s Hospital between January 2010 and December 2014. Demographic information and biochemical parameters were systematically compiled, and the diagnosis of NAFLD was established through ultrasonographic evidence.

**Results:**

This study cohort included 16,172 non-obese participants with a 5-year follow-up, among whom 2,322 (14.36%) developed NAFLD. The disparity between TyG index quartiles in the accumulative incidence of new-onset NAFLD was distinct, with an increasing risk of new-onset NAFLD as the TyG index increased. Participants in highest quartile exhibited the maximum risk of NAFLD. In the fully adjusted model 3, the hazard ratios for NAFLD in Q2, Q3, and Q4 were 2.15 (1.62, 2.87), 2.89 (2.20, 3.80) and 4.58 (3.48, 6.02), respectively. Meanwhile, the TyG index and NAFLD risk showed a highly significant overall correlation (*p* < 0.0001) and nonlinearity (*p* < 0.0001) according to the limited cubic splines. In subgroup analysis, a significant interaction was noted between new-onset NAFLD and SBP (<140 mmHg vs. ≥140 mmHg; P for interaction = 0.0114). The SBP < 140 mmHg subgroup demonstrated an enhanced TyG index influence on NAFLD risk (HR = 2.83, 95% CI: 2.48–3.23, *p* < 0.0001).

**Conclusion:**

The TyG index serves as a straightforward instrument for assessing NAFLD risk in non-obese individuals, enabling prompt identification and management in this population segment.

## 1 Introduction

Non-alcoholic fatty liver disease (NAFLD) constitutes a predominant etiology of chronic hepatic metabolic disorders, characterized by hepatic steatosis as demonstrated through radiological or histopathological assessment, occurring without exogenous etiologies such as significant alcohol intake or pharmacological intervention. Nowadays, the prevalence of NAFLD is as high as 25% worldwide, with nearly one billion people affected (1). According to epidemiological results epidemiological results, the prevalence is relatively high among Asian populations, at around 27% (2, 3). NAFLD and its progressive form, NASH, affect approximately 30 and 5% of the US population, respectively (1). Given the association of NAFLD with elevated risks of cardiovascular and chronic renal diseases, it significantly escalates global morbidity and mortality rates. Moreover, NAFLD is rapidly emerging as a predominant cause of end-stage liver disease and necessitating liver transplantation (4, 5).

Studies have shown that age, obesity, dyslipidemia, and insulin resistance are important risk factors for NAFLD (6). However, a considerable segment of the NAFLD population maintains normal or even below-average Body Mass Indexes (BMIs). Roughly 40% of people with NAFLD worldwide are non-obese (BMI < 25 kg/m^2), while 20% are classified as thin (BMI < 23 kg/m^2^), with nearly 20% being lean individuals (BMI < 23 kg/m^2^) (7). Moreover, non-obese individuals diagnosed with NAFLD exhibit a heightened susceptibility to severe liver diseases such as liver failure, hepatocellular carcinoma, and cirrhosis, as well as cardiovascular diseases, compared with overweight patients (8, 9). Therefore, a simple, effective, non-invasive evaluation index is needed to identify NAFLD in non-obese population.

The etiology of NAFLD is closely associated with insulin resistance (IR), particularly among patients with normal BMI and waist circumference (WC) (10). The Triglyceride Glucose (TyG) index, recently acknowledged as a novel biomarker for detecting IR, is calculated through the product of fasting plasma glucose (FPG) and fasting triglycerides (TG) levels (11). Limited investigations have elucidated the efficacy of the TyG index in prognosticating NAFLD risk (12, 13), but the majority of these studies were cross-sectional and lacked a focus on the non-obese population.

Therefore, to explore the prognostic capacity of the TyG index regarding NAFLD risk among non-obese individuals, our investigation delineated the longitudinal association between the TyG index and the incidence of new-onset NAFLD within the non-obese cohort.

## 2 Methods

### 2.1 Participants and study design

This longitudinal, retrospective cohort study, approved by the Ethics Committee of Wenzhou People’s Hospital, targeted non-obese individuals (BMI ≤ 25 kg/m^2^, LDL ≤ 3.12 mmol/L) selected from health screenings at the Wenzhou Medical Center of the Wenzhou People’s Hospital between January 2010 and December 2014. Exclusions applied to individuals with high alcohol intake, those on antihypertensive, antidiabetic, or lipid-lowering medications, and those with a history of viral hepatitis, autoimmune hepatitis, or other known etiologies of chronic liver disease. Lost to follow-up and incomplete data were also excluded. The methodology and main outcomes have been previously published in the study by Sun et al. (14).

### 2.2 Data collection

Baseline demographics and an extensive clinical profile were documented for each participant. BMI, an indicator of body fat, as an index of adiposity derived from height and weight. Blood pressure readings were obtained in a calm setting with the participant seated. A biochemical panel assessing metabolic health included markers for liver function, kidney function, fasting plasma glucose (FPG), and lipid profile, all of which were analyzed using standard methods on an automated Abbott AxSYM analyzer. The formula ln(fasting triglycerides (mg/dL) × FPG (mg/dL)/2) was used to create the TyG index (15).

### 2.3 Diagnosis of NAFLD

Abdominal ultrasonography, utilized abdominal ultrasonography by certified technicians adhering to the Chinese Liver Disease Association’s diagnostic standards for NAFLD detection (14). This condition was identified by a decreased far-field echo and an enhanced near-field echo in the liver region, which was more pronounced than in the kidney or spleen areas.

Diagnostic criteria encompassed a spectrum of manifestations, from obscured intrahepatic ductal architecture and varying degrees of hepatic enlargement with a softer, rounded contour to uniformly diminished yet normally patterned blood flow signals and inadequate or non-existent visualization of the right hepatic lobe’s outer layer and the diaphragm. Hepatic ultrasound evaluations were carried out blindly to determine the achievement of the study’s outcome objectives.

### 2.4 Statistical analysis

Statistical analyses were executed utilizing the R software environment (Version 4.3.2; The R Foundation; accessible at http://www.R-project.org). Participants were stratified by the TyG index into quartiles in an ascending sequence for the subsequent comparison of attributes among these groups. For variables adhering to a normal distribution and measured on a metric scale, we depicted the data using mean ± standard deviation. Conversely, variables characterized by a non-normal, skewed distribution were presented through the median and interquartile range. Analytical comparisons between multiple groups, where data exhibited normal distribution, were conducted utilizing one-way analysis of variance (ANOVA), while the Kruskal-Wallis rank test was employed for analyses under skewed distribution scenarios. For categorical variables, presented as percentages, the chi-square or Fisher’s exact tests were applied to assess the differences. This methodical approach facilitated a nuanced evaluation of the distribution and impact of the TyG index across participant quartiles. The follow-up duration for each participant was calculated from the baseline visit date to the earlier of two endpoints: the conclusion of the follow-up period or the confirmation of a NAFLD diagnosis.

The cumulative risk of new-onset NAFLD was described by the Kaplan-Meier curve, and the cumulative risk of different TyG groups was compared with the log-rank test. Subsequently, Cox proportional hazards models were employed to explore the association between the TyG index and the risk of developing NAFLD. The initial model (Model 1) remained unadjusted, whereas Model 2 incorporated adjustments for age and sex. The determination of covariates for inclusion or removal from the primary model hinged upon their effect on the regression coefficients for TyG quartiles, with shifts exceeding 10% marking them as pivotal for adjustments. Variates including sex, age, BMI, HDL, LDL, TC, DBIL were finally selected into Model 3. Analyses of linear trends were performed by treating TyG index quartiles as ordinal variables in a continuous framework. Subgroup analysis was conducted to estimate the consistency of the effect in different groups including sex, age (<60, ≥60 years), BMI (<18.5, ≥18.5 kg/m^2^) and systolic blood pressure (SBP) (<140, ≥140 mmHg). Statistical significance was determined based on two-tailed *p* values, with values less than 0.05 considered to be statistically significant.

## 3 Results

### 3.1 Baseline characteristics of the participants

The study cohort comprised a total of 16,172 non-obese participants. Table 1 presents the foundational attributes of the cohort, comprising 8,483 (52.45%) males and 7,689 (47.55%) females, with a mean participant age of 43.23 ± 14.96. After an average follow-up of 33.6(13.7) months, 2,322(14.36%) participants were diagnosed with NAFLD.

**Table 1:** Baseline characteristics of the participants in TyG quartiles.

TyG quartile	Q1	Q2	Q3	Q4	*p*-value
N	4,040	4,046	4,042	4,044	
Male, *N* (%)	1918 (47.5%)	2095 (51.8%)	2,146 (53.1%)	2,324 (57.5%)	<0.001
AGE, mean ± SD	41.5 ± 14.1	43.1 ± 15.0	43.6 ± 15.1	44.7 ± 15.4	<0.001
TyG, mean ± SD	7.8 ± 0.2	8.2 ± 0.1	8.6 ± 0.1	9.1 ± 0.4	<0.001
BMI, mean ± SD	20.4 ± 1.9	21.1 ± 2.0	21.6 ± 2.0	22.4 ± 1.8	<0.001
SBP, mean ± SD	113.6 ± 14.1	118.6 ± 16.0	122.8 ± 16.4	127.9 ± 16.8	<0.001
DBP, mean ± SD	68.8 ± 9.3	71.6 ± 9.6	73.7 ± 10.1	77.2 ± 10.5	<0.001
ALP (U/L), median (IQR)	61.0 (51.0–74.0)	67.0 (56.0–81.0)	72.0 (59.0–86.0)	75.0 (64.0–90.0)	<0.001
GGT (U/L), median (IQR)	17.0 (14.0–21.0)	19.0 (15.0–25.0)	22.0 (18.0–31.0)	30.0 (22.0–47.0)	<0.001
ALT (U/L), median (IQR)	14.0 (11.0–18.0)	15.0 (12.0–21.0)	17.0 (13.0–23.0)	20.0 (15.0–28.0)	<0.001
AST (U/L), median (IQR)	20.0 (17.0–23.0)	21.0 (18.0–24.0)	22.0 (19.0–25.0)	23.0 (20.0–27.0)	<0.001
ALB (g/L), mean ± SD	44.2 ± 2.6	44.2 ± 2.8	44.5 ± 2.8	44.7 ± 2.7	<0.001
TB (μmol/L), mean ± SD	12.0 ± 5.0	12.1 ± 4.8	12.2 ± 4.8	12.2 ± 5.2	0.333
DBIL (μmol/L), median (IQR)	2.2 (1.6–3.0)	2.1 (1.6–2.8)	2.1 (1.5–2.8)	1.8 (1.3–2.5)	<0.001
BUN (mmol/L), median (IQR)	4.3 (3.6–5.2)	4.3 (3.6–5.2)	4.5 (3.7–5.4)	4.5 (3.7–5.4)	<0.001
CR (μmol/L), median (IQR)	67.0 (59.0–77.0)	73.0 (63.0–88.0)	80.0 (67.0–94.0)	85.0 (72.0–96.0)	<0.001
UA (μmol/L), median (IQR)	228.0 (190.0–276.0)	253.0 (206.0–313.0)	283.5 (230.0–343.0)	323.0 (265.0–384.0)	<0.001
GLU (mmol/L), mean ± SD	4.8 ± 0.4	5.0 ± 0.5	5.2 ± 0.6	5.5 ± 1.2	<0.001
TC (mmol/L), mean ± SD	4.4 ± 0.7	4.5 ± 0.7	4.7 ± 0.7	4.9 ± 0.8	<0.001
TG (mmol/L), median (IQR)	0.7 (0.6–0.7)	0.9 (0.9–1.0)	1.3 (1.2–1.4)	2.0 (1.7–2.5)	<0.001
HDL-c (mmol/L), mean ± SD	1.6 ± 0.4	1.5 ± 0.3	1.4 ± 0.3	1.3 ± 0.3	<0.001
LDL-c (mmol/L), mean ± SD	2.1 ± 0.4	2.2 ± 0.4	2.4 ± 0.4	2.4 ± 0.5	<0.001
NAFLD, N(%)	105 (2.6%)	292 (7.2%)	567 (14.0%)	1,358 (33.6%)	<0.001

TyG, triglyceride glucose; BMI, body mass index; SBP, systolic blood pressure; DBP, diastolic blood pressure; ALP, alkaline phosphatase; GGT, gamma glutamyl transferase; ALT, alanine aminotransferase; AST, aspartate aminotransferase; ALB, albumin; TB, total bilirubin; DBIL, direct bilirubin; BUN, blood urea nitrogen; CR, serum creatinine; UA, uric acid; GLU, glucose; TC, total cholesterol; TG, total triglycerides; HDL-c, high-density lipoprotein cholesterol; LDL-c, low-density lipoprotein cholesterol; NAFLD, non-alcoholic fatty liver disease.

In higher quartiles of TyG, a notably higher rate of new-onset NAFLD was observed (*p* < 0.001). Furthermore, individuals in these higher TyG groups tended to be male and had advanced age as well as increased measurements of higher BMI, blood pressure (SBP, diastolic blood pressure (DBP)), liver enzymes (including ALP, GGT, ALT, AST), ALB, BUN, CR, UA, GLU, TC, TG, LDL-C, whereas DBIL and HDL-c levels were lower in this group compared to those in the lower TyG index groups (all *p* < 0.001). However, TB levels did not show a statistically significant variation among the different groups (*p* > 0.05).

### 3.2 Kaplan-Meier curve analysis of NAFLD incidence by TyG index quartiles

**Figure 1:**
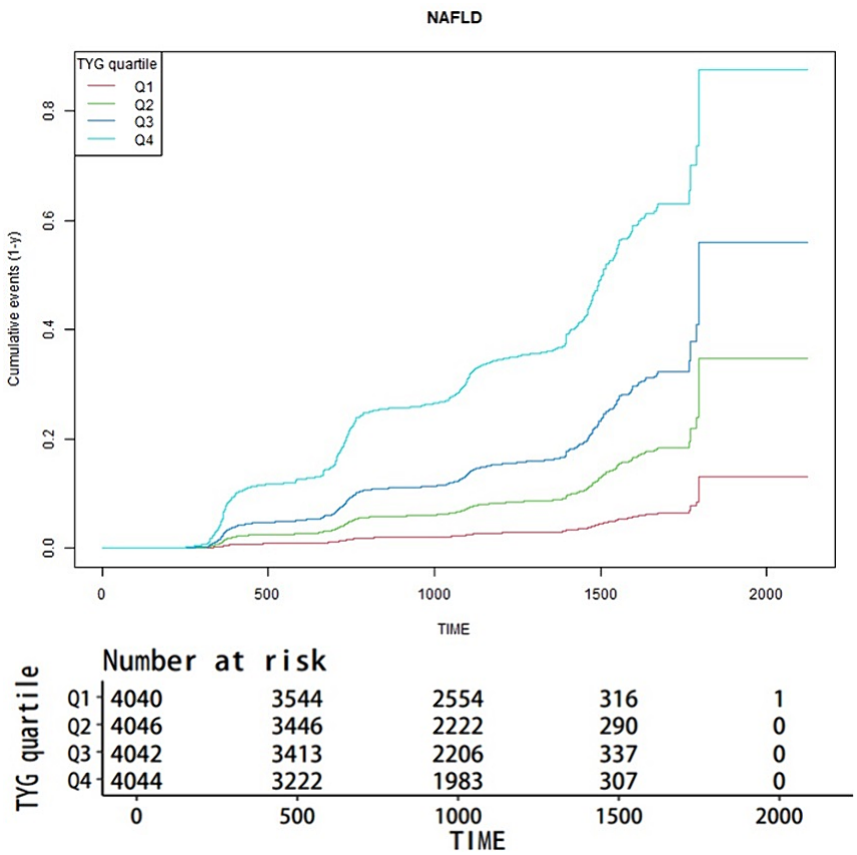
Kaplan-Meier estimator of new-onset NAFLD by TyG quartiles.

Figure 1 illustrates the aggregate occurrence of newly diagnosed NAFLD via Kaplan-Meier curves, stratified by TyG index quartiles. A pronounced disparity (*p* < 0.001) was observed in NAFLD risk across different TyG index levels, with an ascending TyG index correlating to an incremental prevalence of NAFLD. Notably, the uppermost quartile distinctly exhibited the greatest risk.

### 3.3 Relationship between TyG index and new-onset NAFLD

Table 2 presents the hazard ratios (HRs) with 95% confidence intervals (CIs) for new-onset NAFLD among participants, stratified by TyG index quartiles. In the initial unadjusted Model 1, the HRs for NAFLD in Q2, Q3, and Q4 were 3.05 (2.44, 3.81), 5.87 (4.77, 7.23), and 14.89 (12.20, 18.16) respectively. The HRs remained comparable in Model 2 even after adjusting for sex and age. In Model 3, which accounted for potential confounders including sex, age, BMI, HDL, LDL, TC, and DBIL, the HRs for the TyG index and NAFLD in Q2, Q3, and Q4 were 2.15 (1.62, 2.87), 2.89 (2.20, 3.80), and 4.58 (3.48, 6.02), respectively.

**Table 2:** Association between TyG groups and incidence of NAFLD.

TyG groups	Model 1	Model 2	Model 3
	HR (95% CI) *p* value		
Total			
Q1	1.0	1.0	1.0
Q2	3.05 (2.44, 3.81) <0.0001	3.03 (2.43, 3.79) <0.0001	2.15 (1.62, 2.87) <0.0001
Q3	5.87 (4.77, 7.23) <0.0001	5.84 (4.74, 7.19) <0.0001	2.89 (2.20, 3.80) <0.0001
Q4	14.89 (12.20, 18.16) <0.0001	14.74 (12.08, 17.98) <0.0001	4.58 (3.48, 6.02) <0.0001
P for trend	2.36 (2.25, 2.47) <0.0001	2.35 (2.24, 2.46) <0.0001	1.56 (1.46, 1.66) <0.0001

Model 1 was not adjusted.

Model 2 was adjusted for sex, age.

Model 3 was adjusted for sex, age, BMI, HDL, LDL, TC, and DBIL*.

* Covariates were strategically added to or subtracted from the foundational model to evaluate their influence on the regression coefficients of TyG index. Those that resulted in a change greater than 10% in the coefficients were recognized as variables requiring adjustment.

### 3.4 TyG and NAFLD connection assessed using restricted cubic spline analysis

**Figure 2:**
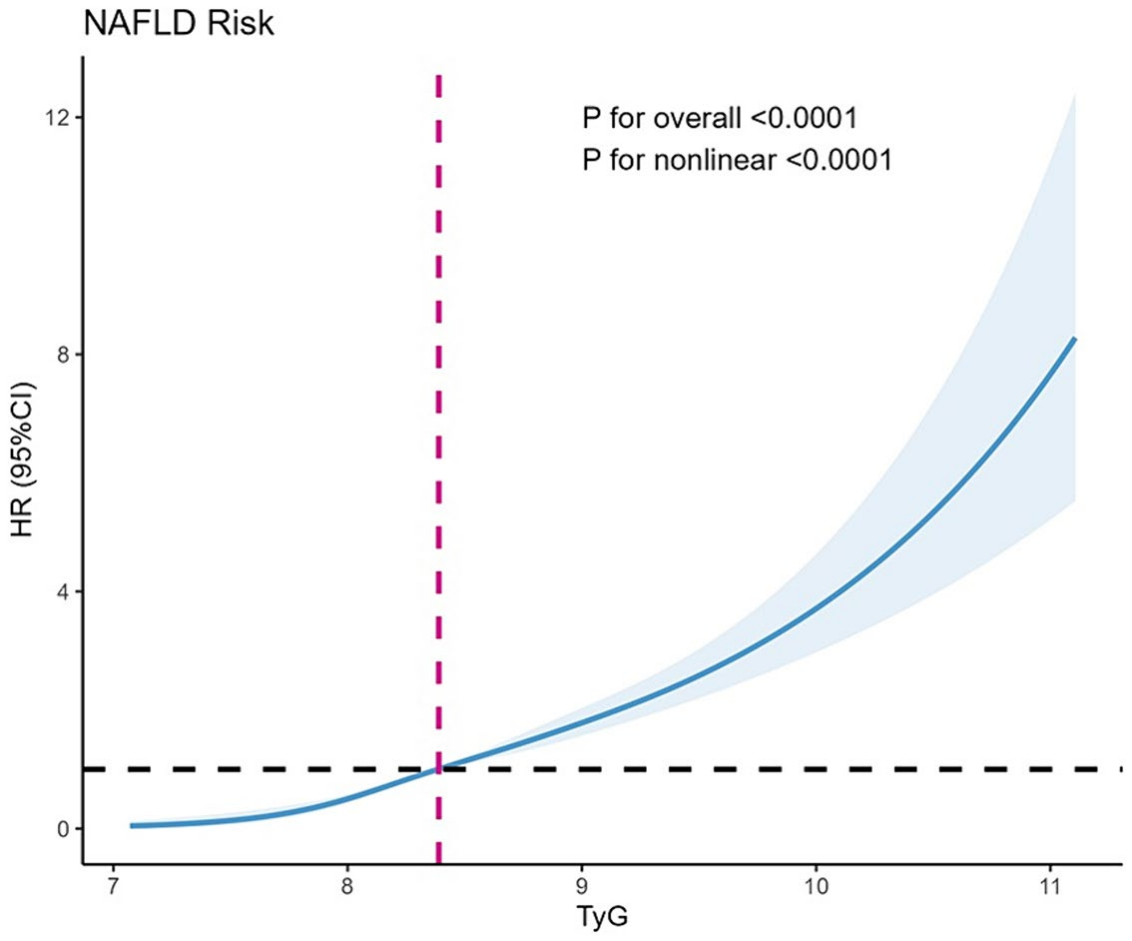
Restricted cubic splines for new onset NAFLD by TyG. The y axis represents HR with the shaded area representing 95% CIs (overall trend, *p* < 0.001; nonlinear trend, *p* < 0.001). Model is adjusted for sex, age, HDL, LDL, DBIL, TC, BMI. TyG, triglyceride glucose; HR, hazard ratio; CI, confidence interval; NAFLD, non-alcoholic fatty liver disease.

To advance the examination of the link between the TyG index and NAFLD susceptibility, a restricted cubic spline (RCS) analysis was utilized to assess the dose–response trajectory correlating TyG levels with NAFLD occurrence. The results obtained from the RCS analysis illustrated a pronounced nonlinear relationship between the TyG index and the risk of new-onset NAFLD (Figure 2). The RCS model highlighted a statistically significant association with an increase in the TyG index correlating to a heightened risk of NAFLD (*p*-value for overall and *p*-value for nonlinearity both <0.0001).

### 3.5 Subgroup analysis for the risk of new-onset NAFLD by TyG index

**Figure 3:**
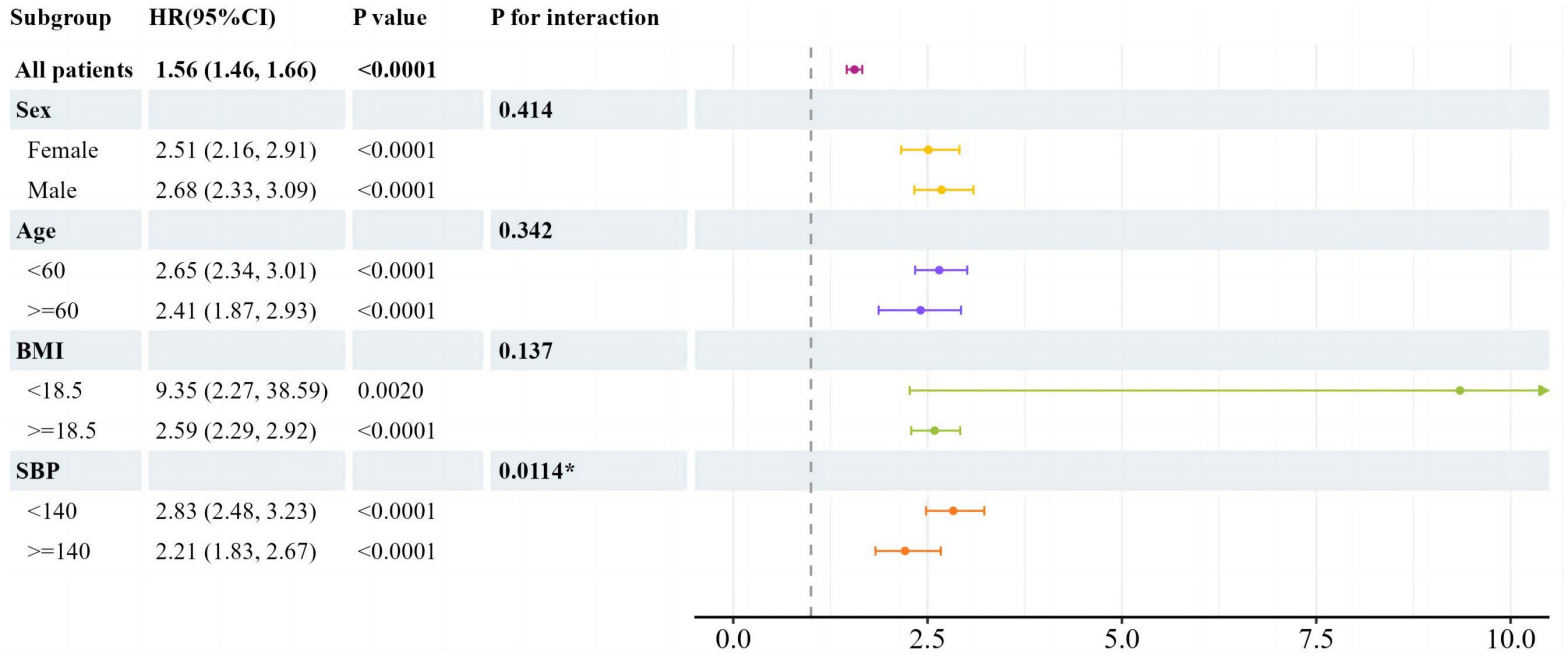
Subgroup analysis of associations between newly onset NAFLD and TyG by univariate cox regression analysis. HR per 1 increase in TyG. TyG, triglyceride glucose; BMI, body mass index; SBP, systolic blood pressure; HR, hazard ratio; CI, confidence interval.

To delve deeper into additional risk factors, we conducted a subgroup analysis focusing on the association between the TyG index and the occurrence of newly diagnosed NAFLD (Figure 3). The analysis was segmented based on sex, age, BMI, and SBP. A notable interaction was detected between SBP and the new-onset NAFLD (P for interaction = 0.0114). For individuals with an SBP below 140 mmHg, the HR was 2.83 (95% CI: 2.48–3.23, *p* < 0.0001). In contrast, for those with an SBP of 140 mmHg or above, the HR was reduced to 2.21 (95% CI: 1.83–2.67, *p* < 0.0001). Within the BMI subgroup, participants with a BMI under 18.5 had an HR of 9.35 (95% CI: 2.27–38.59, *p* = 0.0020), while those with a BMI of 18.5 or above had an HR of 2.59 (95% CI: 2.29–2.92, *p* < 0.0001). However, this difference in BMI subgroups did not show a significant interaction (P for interaction = 0.137). There were no discernible relationships between the TyG index and the age and sex subgroups, as all the interaction *p*-values were above 0.05.

## 4 Discussion

In this longitudinal cohort study, we examined the link between the TyG index and the emergence of new-onset NAFLD among non-obese individuals. The results consistently showed a significant association between higher TyG index values and the occurrence of non-alcoholic fatty liver disease, even when controlling for confounding variables. Apart from variations in SBP, this association persisted uniformly across different subgroups. Notably, an interaction between SBP levels and the onset of NAFLD was identified in the subgroup analysis (P for interaction = 0.0114), revealing that the TyG index offered enhanced prognostic accuracy for identifying NAFLD risk in individuals with non-elevated SBP.

NAFLD, recognized as the most common chronic liver condition, is defined by the excessive buildup of lipids within the liver, impacting individuals regardless of obesity status. Disturbances in glycolipid metabolism play a pivotal role in its progression. Despite limited understanding of NAFLD’s pathogenesis, it is widely accepted that insulin resistance (IR) is closely associated with its development. In 1998, a British researcher introduced the “two-hit theory” of NAFLD for the first time worldwide (16). According to this theory, IR acts as the initial hit, while oxidative stress serves as the second hit. IR promotes the mobilization of peripheral fat and elevates free fatty acid levels in the bloodstream, leading to mitochondrial oxidative overload in hepatocytes and subsequent hepatic lipid accumulation. As research progresses, the “multiple-hit theory” has largely overtaken the “two-hit theory” in explaining the pathogenesis of NAFLD, yet IR remains acknowledged as a crucial factor in the disease’s onset. The review by Reena et al. highlighted the significant link between IR and NAFLD, indicating that individuals with diabetes exhibit a 5-fold higher prevalence of NAFLD compared to non-diabetic persons (17). In their study, Smith et al. showed that the hepatic *de novo* lipogenesis (DNL), a key factor in NAFLD-related steatosis, escalates concurrently with the rise in insulin-resistant glucose metabolism (18). Currently, NAFLD stands as the fastest escalating cause of liver-related mortality and morbidity (19). Given the severity of the disease, it is necessary to identify an effective marker to identify the presence and extent of IR to predict NAFLD, especially in non-obese individuals.

The TyG index is a mathematical model initially proposed by Simental-Mendia et al. It was primarily designed to assess IR in South American populations (15). The index is derived from the measurement of serum triglycerides and fasting glucose levels in patients, making it a suitable tool for large-scale epidemiological screening of populations. Subsequent studies have confirmed that the TyG index is a reliable proxy for insulin resistance that can be used in a variety of settings and for a wide range of racial, ethnic, sex and age groups (11, 20–24). Moreover, in cross-sectional studies conducted by Luis et al., it was demonstrated that the TyG index was negatively correlated with insulin secretion in young adults who were of normal weight, highlighting the index’s potential as a comprehensive indicator of metabolic dysfunction (25). Given the significant role of IR in the development of NAFLD, the TyG index, which can determine the degree and existence of IR, holds considerable diagnostic value in identifying NAFLD. Additionally, NAFLD in non-obese individuals can sometimes lead to more adverse outcomes than in obese patients, including a faster progression to cirrhosis (26). Previous studies have demonstrated the association between the TyG index and liver fibrosis (27). Patients with higher TyG index quartiles exhibit an increased prevalence of NAFLD and liver fibrosis (28). The progression of fibrosis occurs more rapidly in lean individuals with NAFLD compared to those with a higher BMI (29). This suggests the potential value of the TyG index in predicting NAFLD in non-obese populations.

Current predictive tools for NAFLD, such as the fatty liver index (FLI), hepatic steatosis index (HSI), and various imaging modalities, have limitations in terms of accessibility, cost, and accuracy, especially in non-obese populations. The FLI and HSI, for instance, often rely on parameters like BMI and waist circumference, which may not accurately capture the risk in individuals with a normal BMI (30, 31). Imaging techniques, while effective, are often costly and not feasible for widespread screening in large populations (32). The TyG index, calculated from routine laboratory measures of fasting plasma glucose and triglycerides, offers a cost-effective and easily accessible alternative. Recent years have witnessed an increase in studies validating the association between the TyG index and NAFLD in the broader population. According to Zheng et al.’s longitudinal research, which included 4,539 Chinese subjects over a nine-year follow-up, the TyG index exhibited enhanced diagnostic accuracy over TG, ALT, and FPG based on ROC curve assessments. Furthermore, Cox regression analyses underscored an independent and positive correlation between the TyG index and the likelihood of developing NAFLD (33). In a longitudinal cohort study, Kim et al. enrolled 52,575 adults and followed them for a median of 5.1 years. They discovered that the risk of NAFLD increased when the TyG index level went from moderate to high, and that the level at which the risk increased appeared to be 8.24 (34). In the cohort study conducted Wang et al., conducted over a follow-up period of 53,481 person-years, those in the highest quartile of baseline TyG index were found to have 2.52-fold higher risk (95% CI, 2.21–2.86) of developing NAFLD compared to those in the lowest quartile (35). In order to investigate the relationship between the TyG index and the prognosis of NAFLD, Liu et al. conducted a cohort study involving 11,424 patients with NAFLD. The study discovered that greater TyG levels were linked to a higher risk of NAFLD development and a worse chance of NAFLD improvement over a median follow-up period of 21 months (36). A cross-sectional study of 4,986 Korean adults compared the TyG index and HOMA-IR as predictors of NAFLD, showing the superiority of the TyG index in this regard (37). Additionally, the TyG-BMI score and NAFLD showed an independent and favorable connection, according to the cross-sectional investigation by Wang et al. (38).

However, the majority of these research on the connection between NAFLD and the TyG index have been conducted on a broad population. Although lean or non-obese people make up roughly 25% of the NAFLD population (39), similar studies focusing on non-obese individuals are scarce. Therefore, this study may provide important clinical evidence that can be used to improve early detection and intervention strategies for NAFLD in this population.

### 4.1 Strengths and limitations

Key features of this study included its prospective framework, extended follow-up duration, substantial cohort size, and focus on a non-obese population. Additionally, robust statistical adjustments were made to reduce confounding factors, ensuring more reliable findings. Nonetheless, there are several restrictions that must be recognized. Firstly, as an observational study, the evidentiary standards are lower, and it cannot establish causal relationships. Secondly, the lack of data on variables such as smoking, alcohol consumption, waist circumference, and hip circumference is a potential limitation that may impact the results. Moreover, the reliance on abdominal ultrasound as the primary diagnostic tool for NAFLD may have missed cases with lower fat content. Furthermore, the study did not differentiate between various pathological states and progression stages of fatty liver, which limited the evaluation of the TyG index for predicting outcomes like liver fibrosis and hepatocellular carcinoma. Future research should aim to address these limitations by incorporating comprehensive data on lifestyle factors, including dietary habits, physical activity, smoking status, and alcohol consumption. Investigating the TyG index’s predictive capability for different stages of fatty liver disease, including liver fibrosis and hepatocellular carcinoma, would provide deeper insights into its clinical utility. Expanding the study to diverse populations and including a broader range of potential confounders will further validate the TyG index as a universal predictive marker for NAFLD. By exploring these directions, future research can build on our findings to offer more robust and comprehensive insights into the relationship between the TyG index and NAFLD.

## Data availability statement

Publicly available datasets were analyzed in this study. This data can be found here: Dryad data repository at http://datadryad.org/ under the doi: 10.5061/dryad.1n6c4.14.

## Ethics statement

The studies involving humans were approved by the Ethics Committee of Affiliated Hospital of Yangzhou University. The studies were conducted in accordance with the local legislation and institutional requirements. Written informed consent for participation was not required from the participants or the participants’ legal guardians/next of kin in accordance with the national legislation and institutional requirements.

## Author contributions

QN: Methodology, Writing – original draft. KZ: Formal analysis, Writing – original draft. JY: Conceptualization, Writing – original draft. CZ: Supervision, Writing – review & editing.

## References

[1] Cotter TG Rinella M. Nonalcoholic fatty liver disease 2020: the state of the disease. Gastroenterology. (2020) 158:1851–64. doi: 10.1053/j.gastro.2020.01.052, PMID: 32061595

[2] Younossi Z Tacke F Arrese M Chander SB Mostafa I Bugianesi E. Global perspectives on nonalcoholic fatty liver disease and nonalcoholic steatohepatitis. Hepatology. (2019) 69:2672–82. doi: 10.1002/hep.30251 30179269

[3] Younossi ZM Koenig AB Abdelatif D Fazel Y Henry L Wymer M. Global epidemiology of nonalcoholic fatty liver disease-meta-analytic assessment of prevalence, incidence, and outcomes. Hepatology. (2016) 64:73–84. doi: 10.1002/hep.28431, PMID: 26707365

[4] Carr RM Oranu A Khungar V. Nonalcoholic fatty liver disease: pathophysiology and management. Gastroenterol Clin N Am. (2016) 45:639–52. doi: 10.1016/j.gtc.2016.07.003, PMID: 27837778 PMC5127277

[5] Cobbina E Akhlaghi F. Non-alcoholic fatty liver disease (nafld) – pathogenesis, classification, and effect on drug metabolizing enzymes and transporters. Drug Metab Rev. (2017) 49:197–211. doi: 10.1080/03602532.2017.1293683, PMID: 28303724 PMC5576152

[6] Pouwels S Sakran N Graham Y Leal A Pintar T Yang W. Non-alcoholic fatty liver disease (nafld): a review of pathophysiology, clinical management and effects of weight loss. BMC Endocr Disord. (2022) 22:63. doi: 10.1186/s12902-022-00980-1 35287643 PMC8919523

[7] Ye Q Zou B Yeo YH Li J Huang DQ Wu Y. Global prevalence, incidence, and outcomes of non-obese or lean non-alcoholic fatty liver disease: a systematic review and meta-analysis. Lancet Gastroenterol Hepatol. (2020) 5:739–52. doi: 10.1016/S2468-1253(20)30077-7 32413340

[8] Yoshitaka H Hamaguchi M Kojima T Fukuda T Ohbora A Fukui M. Nonoverweight nonalcoholic fatty liver disease and incident cardiovascular disease: a post hoc analysis of a cohort study. Medicine (Baltimore). (2017) 96:e6712. doi: 10.1097/MD.0000000000006712, PMID: 28471965 PMC5419911

[9] Phipps M Wattacheril J. Non-alcoholic fatty liver disease (nafld) in non-obese individuals. Frontline Gastroenterol. (2020) 11:478–83. doi: 10.1136/flgastro-2018-101119, PMID: 33101626 PMC7569516

[10] Chang M Shao Z Shen G. Association between triglyceride glucose-related markers and the risk of metabolic-associated fatty liver disease: a cross-sectional study in healthy chinese participants. BMJ Open. (2023) 13:e070189. doi: 10.1136/bmjopen-2022-070189, PMID: 37130686 PMC10163481

[11] Ramdas NV Satheesh P Shenoy MT Kalra S. Triglyceride glucose (tyg) index: a surrogate biomarker of insulin resistance. J Pak Med Assoc. (2022) 72:986–8. doi: 10.47391/JPMA.22-63 35713073

[12] Beran A Ayesh H Mhanna M Wahood W Ghazaleh S Abuhelwa Z. Triglyceride-glucose index for early prediction of nonalcoholic fatty liver disease: a meta-analysis of 121,975 individuals. J Clin Med. (2022) 11:2666. doi: 10.3390/jcm11092666, PMID: 35566790 PMC9102411

[13] Ling Q Chen J Liu X Xu Y Ma J Yu P. The triglyceride and glucose index and risk of nonalcoholic fatty liver disease: a dose-response meta-analysis. Front Endocrinol (Lausanne). (2022) 13:1043169. doi: 10.3389/fendo.2022.1043169, PMID: 36743937 PMC9892833

[14] Sun DQ Wu SJ Liu WY Wang LR Chen YR Zhang DC. Association of low-density lipoprotein cholesterol within the normal range and nafld in the non-obese chinese population: a cross-sectional and longitudinal study. BMJ Open. (2016) 6:e013781. doi: 10.1136/bmjopen-2016-013781, PMID: 27927668 PMC5168665

[15] Simental-Mendía LE Rodríguez-Morán M Guerrero-Romero F. The product of fasting glucose and triglycerides as surrogate for identifying insulin resistance in apparently healthy subjects. Metab Syndr Relat Disord. (2008) 6:299–304. doi: 10.1089/met.2008.0034, PMID: 19067533

[16] Day CP James OFW. Steatohepatitis: a tale of two “hits”? Gastroenterology. (1998) 114:842–5. doi: 10.1016/S0016-5085(98)70599-2 9547102

[17] Khan RS Bril F Cusi K Newsome PN. Modulation of insulin resistance in nonalcoholic fatty liver disease. Hepatology. (2019) 70:711–24. doi: 10.1002/hep.30429 30556145

[18] Smith GI Shankaran M Yoshino M Schweitzer GG Chondronikola M Beals JW. Insulin resistance drives hepatic de novo lipogenesis in nonalcoholic fatty liver disease. J Clin Invest. (2020) 130:1453–60. doi: 10.1172/JCI134165, PMID: 31805015 PMC7269561

[19] Paik JM Golabi P Younossi Y Mishra A Younossi ZM. Changes in the global burden of chronic liver diseases from 2012 to 2017: the growing impact of nafld. Hepatology. (2020) 72:1605–16. doi: 10.1002/hep.31173, PMID: 32043613

[20] Dikaiakou E Vlachopapadopoulou EA Paschou SA Athanasouli F Kafetzi M Panagiotopoulos Ι. Τriglycerides-glucose (tyg) index is a sensitive marker of insulin resistance in greek children and adolescents. Endocrine. (2020) 70:58–64. doi: 10.1007/s12020-020-02374-6, PMID: 32557329

[21] Huang R Cheng Z Jin X Yu X Yu J Guo Y. Usefulness of four surrogate indexes of insulin resistance in middle-aged population in Hefei, China. Ann Med. (2022) 54:622–32. doi: 10.1080/07853890.2022.2039956 35175162 PMC8856080

[22] Lim J Kim J Koo SH Kwon GC. Comparison of triglyceride glucose index, and related parameters to predict insulin resistance in korean adults: an analysis of the 2007–2010 korean national health and nutrition examination survey. PLoS One. (2019) 14:e0212963. doi: 10.1371/journal.pone.0212963, PMID: 30845237 PMC6405083

[23] Reckziegel MB Nepomuceno P Machado T Renner JDP Pohl HH Nogueira-de-Almeida CA. The triglyceride-glucose index as an indicator of insulin resistance and cardiometabolic risk in brazilian adolescents. Arch Endocrinol Metab. (2023) 67:153–61. doi: 10.20945/2359-3997000000506, PMID: 36651702 PMC10689041

[24] Vega-Cárdenas M Flores-Sánchez J Torres-Rodriguez ML Sánchez-Armáss Capello O Vargas-Morales JM Cossío-Torres PE. Distribution of tyg index and homeostasis model assessment insulin resistance for the evaluation of insulin sensitivity on late adolescence in mexicans. Nutr Hosp. (2022) 39:1349–56. doi: 10.20960/nh.04120, PMID: 36373659

[25] Simental-Mendía LE Gómez-Díaz R Wacher NH Guerrero-Romero F. The triglycerides and glucose index is negatively associated with insulin secretion in young adults with normal weight. Horm Metab Res. (2022) 54:33–6. doi: 10.1055/a-1713-7821, PMID: 34986498

[26] Wang AY Dhaliwal J Mouzaki M. Lean non-alcoholic fatty liver disease. Clin Nutr. (2019) 38:975–81. doi: 10.1016/j.clnu.2018.08.008 30466956

[27] Kim MK Kim JH Park K Lee SB Nam JS Kang S. Relationship between the triglyceride glucose index and the presence and fibrosis of nonalcoholic fatty liver disease in korean adults. Diabetes. (2018) 67:612. doi: 10.2337/db18-612-P

[28] Guo W Lu J Qin P Li X Zhu W Wu J. The triglyceride-glucose index is associated with the severity of hepatic steatosis and the presence of liver fibrosis in non-alcoholic fatty liver disease: a cross-sectional study in chinese adults. Lipids Health Dis. (2020) 19:218. doi: 10.1186/s12944-020-01393-6, PMID: 33028338 PMC7541277

[29] Hagstrom H Nasr P Ekstedt M Hammar U Stal P Hultcrantz R. Risk for development of severe liver disease in lean patients with nonalcoholic fatty liver disease: a long-term follow-up study. Hepatol Commun. (2018) 2:48–57. doi: 10.1002/hep4.1124, PMID: 29404512 PMC5776871

[30] Motamed N Sohrabi M Ajdarkosh H Hemmasi G Maadi M Sayeedian FS. Fatty liver index vs waist circumference for predicting non-alcoholic fatty liver disease. World J Gastroenterol. (2016) 22:3023–30. doi: 10.3748/wjg.v22.i10.3023, PMID: 26973398 PMC4779925

[31] Sviklane L Olmane E Dzerve Z Kupcs K Pirags V Sokolovska J. Fatty liver index and hepatic steatosis index for prediction of non-alcoholic fatty liver disease in type 1 diabetes. J Gastroenterol Hepatol. (2018) 33:270–6. doi: 10.1111/jgh.13814, PMID: 28464337

[32] Ajmera V Loomba R. Imaging biomarkers of nafld, Nash, and fibrosis. Mol Metab. (2021) 50:101167. doi: 10.1016/j.molmet.2021.101167, PMID: 33460786 PMC8324681

[33] Zheng R Du Z Wang M Mao Y Mao W. A longitudinal epidemiological study on the triglyceride and glucose index and the incident nonalcoholic fatty liver disease. Lipids Health Dis. (2018) 17:262. doi: 10.1186/s12944-018-0913-3, PMID: 30458848 PMC6247753

[34] Kim KS Hong S Ahn HY Park CY. Triglyceride and glucose index is a simple and easy-to-calculate marker associated with nonalcoholic fatty liver disease. Obesity (Silver Spring). (2022) 30:1279–88. doi: 10.1002/oby.23438, PMID: 35674697

[35] Wang Y Wang J Liu L Yang P Deng S Liu X. Baseline level and change trajectory of the triglyceride-glucose index in relation to the development of nafld: a large population-based cohort study. Front Endocrinol (Lausanne). (2023) 14:1137098. doi: 10.3389/fendo.2023.1137098, PMID: 37223043 PMC10200880

[36] Liu J Guan L Zhao M Li Q Song A Gao L. Association between the triglyceride-glucose index and outcomes of nonalcoholic fatty liver disease: a large-scale health management cohort study. Diabetes Metab Syndr Obes. (2021) 14:2829–39. doi: 10.2147/DMSO.S316864, PMID: 34188506 PMC8232855

[37] Lee SB Kim MK Kang S Park K Kim JH Baik SJ. Triglyceride glucose index is superior to the homeostasis model assessment of insulin resistance for predicting nonalcoholic fatty liver disease in korean adults. Endocrinol Metab (Seoul). (2019) 34:179–86. doi: 10.3803/EnM.2019.34.2.179, PMID: 31257745 PMC6599902

[38] Wang R DAI L Zhong Y Xie G. Usefulness of the triglyceride glucose-body mass index in evaluating nonalcoholic fatty liver disease: insights from a general population. Lipids Health Dis. (2021) 20:77. doi: 10.1186/s12944-021-01506-9, PMID: 34321005 PMC8317400

[39] Vilarinho S Ajmera V Zheng M Loomba R. Emerging role of genomic analysis in clinical evaluation of lean individuals with nafld. Hepatology. (2021) 74:2241–50. doi: 10.1002/hep.32047, PMID: 34233030 PMC8463418

